# Genomics and Pathways Involved in Maize Resistance to *Fusarium* Ear Rot and Kernel Contamination With Fumonisins

**DOI:** 10.3389/fpls.2022.866478

**Published:** 2022-05-02

**Authors:** Ana Cao, María de la Fuente, Noemi Gesteiro, Rogelio Santiago, Rosa Ana Malvar, Ana Butrón

**Affiliations:** ^1^Misión Biológica de Galicia (CSIC), Pontevedra, Spain; ^2^Agrobiología Ambiental, Calidad de Suelos y Plantas (UVIGO), Unidad Asociada a la MBG (CSIC), Pontevedra, Spain

**Keywords:** maize, *Fusarium verticillioides*, fumonisins, resistance, QTL, differentially expressed genes (DEG)

## Abstract

*Fusarium verticillioides* is a causal agent of maize ear rot and produces fumonisins, which are mycotoxins that are toxic to animals and humans. In this study, quantitative trait loci (QTLs) and bulk-segregant RNA-seq approaches were used to uncover genomic regions and pathways involved in resistance to *Fusarium* ear rot (FER) and to fumonisin accumulation in maize kernels. Genomic regions at bins 4.07–4.1, 6–6.01, 6.04–6.05, and 8.05–8.08 were related to FER resistance and/or reduced fumonisin levels in kernels. A comparison of transcriptomes between resistant and susceptible inbred bulks 10 days after inoculation with *F. verticillioides* revealed 364 differentially expressed genes (DEGs). In the resistant inbred bulks, genes involved in sink metabolic processes such as fatty acid and starch biosynthesis were downregulated, as well as those involved in phytosulfokine signaling and many other genes involved in cell division; while genes involved in secondary metabolism and compounds/processes related to resistance were upregulated, especially those related to cell wall biosynthesis/rearrangement and flavonoid biosynthesis. These trends are indicative of a growth–defense trade-off. Among the DEGs, Zm00001d053603, Zm00001d035562, Zm00001d037810, Zm00001d037921, and Zm00001d010840 were polymorphic between resistant and susceptible bulks, were located in the confidence intervals of detected QTLs, and showed large differences in transcript levels between the resistant and susceptible bulks. Thus, they were identified as candidate genes involved in resistance to FER and/or reduced fumonisin accumulation.

## Introduction

*Fusarium verticillioides* has long been known to be a causal agent of maize ear rot but has received more attention since the discovery that fumonisins, the mycotoxins it produces, can accumulate in maize kernels (Gelderblom et al., 1988). Fumonisin consumption can lead to serious disorders in humans and animals; for example, leukoencephalomalacia in horses and pulmonary edema in swine, both are accompanied by injuries to the liver and heart; hepatic necrosis and, eventually, kidney and liver cancers in rodents; impaired liver function in cattle; and reduced growth in poultry (Munkvold and Desjardins, 1997; Marasas, 2001; Voss et al., 2007). In addition, fumonisins were classified as possibly carcinogenic to humans by the International Agency for Research on Cancer (IARC, 2002). Epidemiological studies have suggested a likely relationship between human consumption of fumonisin-contaminated maize and incidence of esophageal cancer and neural tube defects in human embryos (Rheeder et al., 2002; Gelineau-van Waes et al., 2005; Missmer et al., 2006; Sun et al., 2007; Alizadeh et al., 2012).

Natural infection of maize kernels by *F. verticillioides* results in fumonisin accumulation in field-grown maize plants. Environmental conditions during kernel development and drying affect the level of fumonisin contamination at harvest; however, maize genetics may also affect both infection by the pathogen and fumonisin accumulation (Cao et al., 2013; Santiago et al., 2015). Ratings for *Fusarium* ear rot (FER) have extensively been used to estimate kernel infection by *F. verticillioides*. In previous studies, severity of FER has shown significant and moderate to high correlation coefficients with kernel contamination with fumonisins across different environments and maize materials (Bolduan et al., 2009; Eller et al., 2010; Löffler et al., 2010, 2011; Presello et al., 2011; Santiago et al., 2013). Therefore, most studies focused on reducing kernel contamination with fumonisins through maize resistance management have been devoted to uncovering the genetic basis of resistance to FER (Pérez-Brito et al., 2001; Zila et al., 2014; Chen et al., 2016; Ju et al., 2017). However, *F. verticillioides* has been defined as a maize endophyte, and fungal infection is not always symptomatic. Even symptom-less plants can accumulate fumonisin mycotoxins (Bacon and Hinton, 1996; Rodríguez Estrada et al., 2012).

As an alternative to laboratory quantification of fumonisins, which is tedious and expensive, molecular markers have been proposed as valuable tools in breeding programs for reducing kernel contamination with these mycotoxins. Moreover, selection based on phenotype alone could be inefficient because of the complexity of maize resistance to fumonisin accumulation (Eller et al., 2008). This complexity is mainly due to the different performance of genotypes across environments and the relative relevance of non-additive genetic effects (Butron et al., 2006, 2015; Robertson et al., 2006; Padua et al., 2016; Maschietto et al., 2017). Nevertheless, although dominance effects could be important in inheritance of resistance to fumonisin accumulation, the results of several studies on the genetics of resistance suggest that it is better to focus on inbred materials than on hybrids. This is because genetic variation is greater among inbred lines than among hybrids, and additive effects are more stable than dominance effects. Moreover, moderate to high genotypic correlation coefficients for resistance to fumonisin accumulation have been reported between inbred lines and test crosses (Löffler et al., 2011; Hung and Holland, 2012; Padua et al., 2016). The search for quantitative trait loci (QTLs) for resistance to fumonisin accumulation has been only performed on few mapping populations, most of which are bi-parental populations (Robertson-Hoyt et al., 2006; Maschietto et al., 2017; Morales et al., 2019; Samayoa et al., 2019; Gesteiro et al., 2021). In this sense, this study intends to gain additional insight into the genetic basis of resistance to kernel infection by exploring genetic variability in a new mapping population derived from a cross between two inbreds with contrasting values for both FER and fumonisin accumulation in kernels.

Other molecular tools besides DNA markers can be used at the genome-wide level to study the genetic basis of resistance to FER and kernel contamination with fumonisins. Gene expression analyses have been conducted to identify genes involved in different responses between resistant and susceptible maize genotypes to kernel infection by *F. verticillioides*, as described in several reviews (Lanubile et al., 2017; Santiago et al., 2020). These studies have shed light on genes involved in maize kernel, silk, and sheath responses to infection by *F. verticillioides*. However, there has been limited success in discovering genes involved in resistance, because most studies have used unrelated inbreds with contrasting resistance levels (Lanubile et al., 2010, 2012a,^2014^; Campos-Bermudez et al., 2013; Yuan et al., 2013; Wang et al., 2016). In studies using such materials, the chance of detecting false positive associations between genes and resistance is increased, because different genetic backgrounds of inbreds could underlie differential gene expression. Resistance/susceptibility is the result of continuous fungus–plant communication.

Most studies have focused on the early response of maize plants to infection. However, there is evidence that the biosynthesis of secondary metabolites that are further transformed into antifungal compounds is preferentially induced in the late stage of *F. verticillioides* infection (Wang et al., 2016). In addition, Maschietto et al. (2015) demonstrated that plant resistance responses were mounted between 7 and 14-days post-inoculation (dpi). In this study, we used a bulk-segregant RNA-seq (BSR) approach to compare different inbred pools 10 dpi, since it would allow us to simultaneously identify differentially expressed genes (DEGs) and polymorphic DNA markers related to fumonisin accumulation (Du et al., 2017; Lin et al., 2021).

## Materials and Methods

### Quantitative Trait Loci Mapping for *Fusarium* Ear Rot and Fumonisin Content

A linkage mapping population of 144 recombinant inbred lines (RILs) derived from a cross between the European flint inbred line EP42 and the American dent inbred line A637 was used for QTL mapping for FER and fumonisin content in kernels. EP42 is susceptible to FER and fumonisin contamination, while A637 is partially resistant to both. RIL genotypes at 121 simple sequence repeat (SSR) markers and the population linkage map have been previously published by Samayoa et al. (2014).

The 144 RILs were evaluated in Pontevedra (42°24′N, 8°38′W, and 20 m above sea level), Spain in 2016 and 2017 using a 12 × 12 lattice design with two replications. Trials were hand-planted, and each experimental plot consisted of one row spaced 0.8 m apart from the other row with 13 one-kernel hills spaced 0.18 m apart. Evaluations were performed under artificial inoculation with *F. verticillioides*. Five plants of each plot were inoculated in August 12th and 8th in 2016 and 2017, respectively. In each plot, individual ears with still fresh silks were chosen to be inoculated with 2 ml of a spore suspension (10^6^ spores/ml) of a characterized toxigenic isolate of *F. verticillioides* (Cao et al., 2014). The inoculum was injected into the center of the ear using a four-needle vaccinator that perforated the husks and injured three to four kernels (Reid et al., 1996).

The inoculated ears from each plot were collected 2 months after inoculation and were individually rated for FER using a 7-point scale (1 = no visible disease symptoms, 2 = 1–3%, 3 = 4–10%, 4 = 11–25%, 5 = 26–50%, 6 = 51–75%, and 7 = 76–100% of kernels exhibiting visual symptoms of infection) devised by Reid and Zhu (2005). Then, the inoculated ears from each plot were dried at 35°C for 1 week and shelled, and a kernel sample of approximately 50 g was taken and stored at 4°C until chemical analyses for fumonisin quantification were performed. The kernel samples were ground through a 0.75-mm screen in a Pulverisette 14 rotor mill (Fritsch GmbH, Oberstein, Germany), and total fumonisin (fumonisins B_1_, B_2_, and B_3_) quantification was performed using a commercial ELISA kit (R-Biopharm Rhône Ltd., Glasgow, Scotland, United Kingdom, at the Food Technology Department of the University of Lleida, Spain. Test recovery rate was approximately 60%; assay variation coefficient was approximately 8%; specificities for B_1_, B_2_, and B_3_ were 100%, around 40%, and almost 100%, respectively, and detection limit was 0.025 mg kg^–1^. Extraction and preparation of samples, as well as test performance, were carried out as described in the commercial kits.

Phenotypic data were analyzed using a mixed model with the PROC MIXED procedure of the SAS statistical package (SAS Institute Inc, 2011). Years, replications, blocks within replications, and years and RILs were considered as random effects. Heritabilities (${\hat{h}}^{2}$) across environments were estimated for each trait on a family mean basis as described previously by Holland et al. (2003). The genetic (*r*_*g*_) and phenotypic (*r*_*p*_) correlations between traits were computed following Holland (2006). QTL analyses were performed with best linear unbiased predictor (BLUP) values of the RILs. Log scaling of BLUPs for fumonisin content was conducted to reduce the effect of large peaks in the data analysis (Xi et al., 2014).

The QTL analysis for FER and fumonisin content was conducted with the 144 RIL using the software PlabMQTL (Utz, 2012). The composite interval mapping (CIM) approach was used for QTL detection and to estimate QTL effects. Additive and epistatic (only for fumonisin content) effects were included in the model. Empirical LOD thresholds of 2.43, 3.83, and 1.93 were established to declare significant a putative QTL for days to silking, fumonisin content, and FER, respectively, according to executed permutation tests with 1,000 random reshuffles assuming an experiment-wise error of 0.3. A fivefold cross validation (CV/G) approach was employed for each trait using 1,000 CV runs to determine the frequency of QTL detection (Utz et al., 2000).

### Bulk-Segregant RNA-Seq

Four RILs with the lowest values (resistant) for fumonisin content (10–15 μg/g) and FER (∼2) along with four RILs with the highest values (susceptible, 55–75 μg/g for fumonisin content and ∼4 for FER) were tested in 2018. Fifteen seeds from each RIL were sown in a single row of 3.5 m, with the distance between adjacent rows being 1 m. The RILs were arranged in four pairs, each pair formed with one resistant and one susceptible RIL. RILs in each pair were planted in adjacent rows in order to minimize the contribution of plot differences to gene expression variations. The day on which each plant showed silks was recorded, and the plant was self-crossed; 15 days later, the main ear of the plant was inoculated as previously described. Ears were individually collected 10 days after inoculation; undamaged immature kernels around the inoculation point were removed and immediately stored in a Falcon tube, kept in liquid nitrogen, and stored at −80°C until RNA extraction.

Three replicates (ears) were used for each RIL. Twenty-four sample RNA extractions were performed using Maxwell^®^ 16 LEV Plant RNA Kit with TRIzol in Maxwell^®^ 16 Research Instrument (Promega, Madison, WI, United States). An RNA-seq analysis was conducted using bulks of resistant and susceptible RILs. Each of three resistant RNA bulks were made mixing equal amounts of one-ear RNA from each resistant RIL; and different ears from each RIL were assigned to different bulks. Three susceptible RNA bulks were made in a similar way. Therefore, six libraries were constructed, three for each bulk (resistant and susceptible). Bulk samples were barcoded and prepared for sequencing at Novogene Co., Ltd., where 150-bp paired-end (PE) reads were obtained on Illumina 1.9.

Quality control of Illumina reads was performed with Trimmomatic (Bolger et al., 2014) by removing poor quality reads and trimming poor quality bases. The percentages of retained reads after quality control ranged from 93.9 to 95.3% (Supplementary Table 1). Gene expression was quantified using Kallisto (Bray et al., 2016), whose algorithm uses a pseudo alignment approach to speed up the alignment procedure. The “pseudo alignment” approach makes it possible to quantify reads without recording actual alignments. The bootstrap resampling option to estimate count variability and the accuracy of quantification of each transcript per sample was set to 100. A DEG analysis was performed with Sleuth (Pimentel et al., 2017) by Wald Test to obtain Benjamini–Hochberg false discovery rate (FDR)-corrected *p*-values for each gene. DEGs were considered as those with FDR adjusted *p*-value < 0.05. The EnhancedVolcano R package was used to generate a volcano plot for gene expression (Blighe et al., 2021). To validate the RNA-seq results, the expression levels of seven genes were determined by qRT-PCR. cDNA was synthesized using Go Script Reverse Transcription System (Promega, Madison, WI, United States) following the manufacturer’s instructions and finally diluted 1:2 with nucleotide-free water. The primer sequences used for qPCR of target genes were designed based on gene sequences published in Maize Genomic Database using PRIMER 3 (Rozen and Skaletsky, 2000) and are listed in Supplementary Table 2. Real-time PCR was carried out using SYBR Green Reagents and BrightGREEN 2X qPCR master mix-low ROX (Applied Biological Materials, Richmond, BC, Canada), and three technical replicates were carried out to estimate variations due to the experimental procedure. The reaction conditions were as follows: 94°C for 1 min, followed by 38 cycles of 95°C for 10 s, 58°C for 10 s, and 72C° for 15 s. After amplification, the LinReg PCR V12.17 software (Ruijter et al., 2009; Tuomi et al., 2010) was used to calculate the efficiencies for each gene from the slopes of the standard curves. Three housekeeping genes were also amplified, and *Gap C* was chosen as the internal control to normalize expression data once results from the three internal controls were compared using the BestKeeper software to normalize the data (Pfaffl et al., 2004). The relative expressions for each gene were calculated following the mathematical model proposed by Pfaffl (2001) for relative quantification in RT-PCR.

### Functional Enrichment

Gene Ontology (GO) terms search for each DEG and functional enrichment were performed by combining results of the PANNZER (Koskinen et al., 2015) and ShinyGO v0.61 (Ge et al., 2020) web tools^1^; both programs were run with default parameters. Functions showing FDR-corrected *p*-values < 0.05 were considered enriched. The online search tool STRING was used for the retrieval of interacting genes^2^ (Version 11.0) (Szklarczyk et al., 2019) in order to find possible protein–protein interaction networks among the DEGs. High confidence interactions were only considered (interaction score >0.7).

### Polymorphisms in Differentially Expressed Genes

The variant calling and Mpileup commands of the bcftools package (Li et al., 2009) were used to detect single nucleotide polymorphisms (SNPs) in the DEGs, which were filtered by quality score (>20) and a minimum depth read of 8. Only SNPs genotyped in all of the six samples were retained. Genomic regions containing DEGs with SNPs for which different bases were fixed in the resistant and susceptible bulks and/or INDELs with presence/absence in all the resistant bulk replicates and absence/presence in all the susceptible bulk replicates were considered as involved in resistance. In addition, polymorphic DEGs between the resistant and susceptible RILs located in the confidence intervals of the QTLs mapped were highlighted.

## Results

### Quantitative Trait Loci for *Fusarium* Ear Rot and Kernel Fumonisin Content

Genotypic variability among the RILs was significant for days to silking, FER, and fumonisin content, while the genotype × year interaction was only significant for days to silking and FER (Supplementary Table 3). Histograms of RIL distributions for each trait are shown in Supplementary Figure 1, as well as the intervals where the RIL parents (A637 and EP42) were located and the heritability estimates for each trait on a mean basis. For each trait, many RILs showed phenotypic values higher or lower than those of the best and worst parents, respectively. Heritability estimates were always significantly different from zero. The mean heritability estimates ranged from 0.63 to 0.86.

The phenotypic correlation coefficient between FER and fumonisin content was moderate (*r* = 0.59 ± 0.04), but the genotypic correlation between both traits was high (0.85 ± 0.14). No significant genotypic correlation was found between FER and days to silking (0.1 ± 0.012), but the correlation coefficient between days to silking and fumonisin content, although low, was positive and significant (0.28 ± 0.12).

Models with two, three, and two QTL explained approximately 40, 30, and 17% of the genetic variation in days to silking, FER, and fumonisin content, respectively (Table 1). Additive models were appropriate for days to silking and FER, but significant additive and additive–additive effects were detected for QTLs for fumonisin content. QTLs for FER were detected at bins 4.07–4.1, 6–6.01, and 8.05–8.07, and those for fumonisin content were detected at bins 6.04–6.05 and 8.07–8.08. QTLs for fumonisin content at 8.07–8.08 clearly co-located with QTLs for FER at 8.05–8.07. Individually, A637 alleles at all QTLs reduced FER and fumonisin content, except at the QTL for FER at 4.08. However, the epistatic interaction between A637 alleles at the two QTLs for fumonisin content had positive and significant effects on that trait. No co-localization between QTLs for days to silking and fumonisin content was detected, although the genotypic correlation coefficient between these two traits was significantly different from zero. Although the genotype × environment interaction was significant for days to silking and FER, the QTLs detected showed similar or at least proportional effects on both years. The QTLs for days to silking at bins 6–6.02 and 8.04–8.05 and the QTLs for FER at bins 4.07–4.1 and 8.05–8.07 were identified in a moderate to high percentage of cross-validation runs. However, the QTLs at bin 6.00–6.01 for FER and QTLs for fumonisin content were less reliable, because they were identified in a low percentage of cross-validation runs.

**TABLE 1:** Location of quantitative trait loci (QTLs) mapped in the recombinant inbred line (RIL) population derived from EP42 × A637 for days to silking (DS), *Fusarium* ear rot (FER), and kernel fumonisin content (Fum) under inoculation with *Fusarium verticillioides*^1^.

		Position				Effect			Variance explained (%)			
Trait	Bin	Distance (cM)	95% CI (cM)	Left marker	LOD	Average	2016	2017	*R* ^2^	*p*	Model	CV
DS	6.00–6.02	24	12–34	bnlg1371	4.64	−1.232**	−1.383	−1.232	11.42	13.3		0.89
	8.04–8.05	89	83–95	umc1858	9.85	1.896**	2.257	1.896	23.28	27.1		1.00
											40.4	
FER	4.07–4.10	146	124–168	umc1051	2.48	0.158**	0.231	0.108	7.44	11.4		0.56
	6.00–6.01	0	0–24	bnlg161	2.14	−0.121**	−0.202	−0.070	5.71	8.8		0.33
	8.05–8.07	130	111–149	bnlg240	2.88	−0.162**	−0.290	−0.070	6.66	10.2		0.77
											30.4	
Fum	6.04–6.05	90	78–102	bnlg1154	4.89	−5.442**	−4.453	−4.732	5.50	8.7		0.30
	8.07–8.08	153	139–167	umc1055	3.88	−3.356*	−3.820	−3.081	2.20	3.5		0.17
	6.04–6.05 × 8.07–8.08					4.219*			2.80	4.4		
											16.6	

*, **Significant at 0.05 and 0.01 probability level, respectively; ^1^Quantitative trait loci effects were estimated as the difference between the phenotypic values of the homozygous for A637 and EP42 alleles; R^2^ = the adjusted proportion of phenotypic variance explained by each significant QTL or QTL interaction; p = the adjusted proportion of the genetic variance explained by each QTL or QTL interaction; and model = the adjusted proportion of the genetic variance explained by the final QTL model.

### RNA Sequencing

A total of ∼371 million PE reads were generated; the average for each sample analyzed was approximately 62 million reads (Supplementary Table 1). After filtering, ∼365 million reads were retained, ranging from 48 to 66 million reads for each sample. Out of the 44,301 maize genes of the B73 RefGen_v4, 26, 864 genes were expressed in at least one RIL bulk (Supplementary Data Sheet 1).

### Differential Gene Expression

**FIGURE 1:**
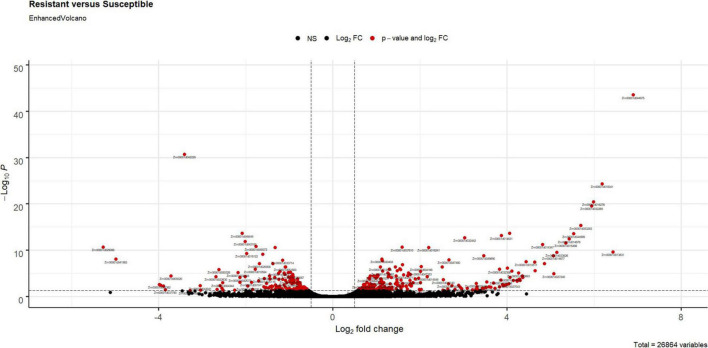
Volcano plot for gene expression in resistant inbred bulks compared to susceptible inbred bulks using the EnhancedVolcano R package (https://github.com/kevinblighe/EnhancedVolcano). The dots located in the positive area stand for genes expressed higher in resistant inbreds, and dots located in the negative area stand for genes expressed higher in susceptible inbreds. As shown in graphic symbol, red stands for expression fold changes higher than 1.5-fold and adjusted *p*-value < 0.05; black stands for no differentially expressed genes (adjusted *p*-value > 0.05 or fold change smaller than 1.5).

Comparison of transcript counts between the resistant and susceptible RIL bulks revealed 364 DEGs (Supplementary Data Sheet 1); the Log_2_ of -fold change values ranged from −5.27 to 6.89 (Supplementary Data Sheet 1 and Figure 1). In the resistant RILs, 212 and 152 genes were upregulated and downregulated, respectively, compared with susceptible RILs. Most of the DEGs (87.9%) were annotated with information in the NCBI and UniProt non-redundant protein databases (Supplementary Data Sheet 2). In a GO enrichment analysis, none of the gene categories were significantly enriched with DEGs (corrected *p*-value < 0.05) (Supplementary Data Sheet 2).

**FIGURE 2:**
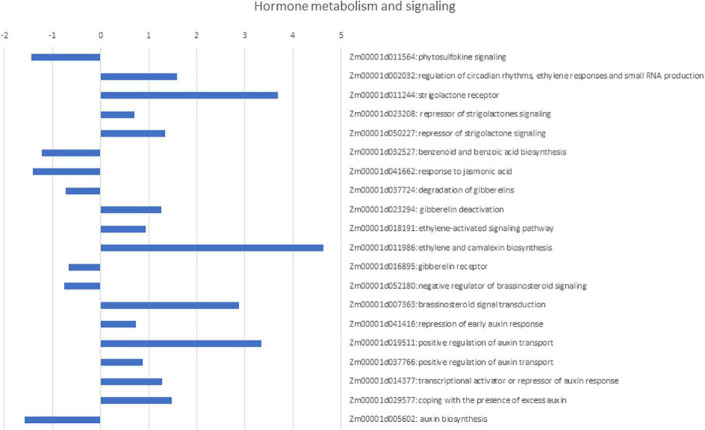
Expression Log_2_ fold change between inbred bulks resistant and susceptible to *Fusarium* ear rot and kernel contamination with fumonisins for differentially expressed genes (DEGs) related to hormone metabolism and signaling.

**FIGURE 3:**
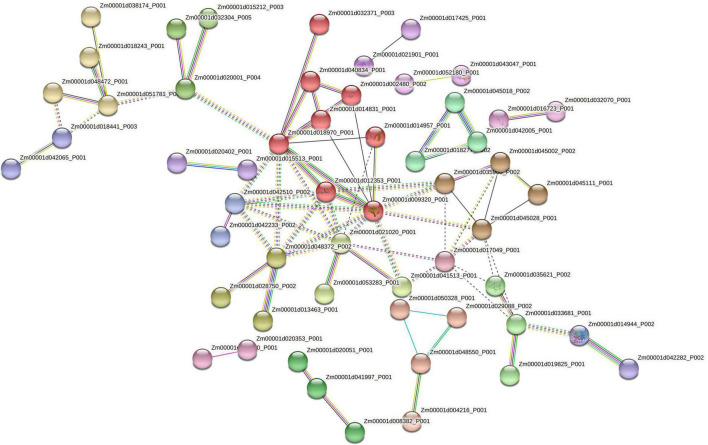
High-confidence protein–protein interaction networks (interaction score >0.7) among DEGs using the online search tool STRING for the retrieval of interacting genes (https://string-db.org/) (Version 11.0) (Szklarczyk et al., 2019).

Two genes, Zm00001d018341 (encoding a protein probably involved in splicing) and Zm00001d044975 (encoding a regulator of the flavonoid pathway), were only expressed in the resistant RILs, while Zm00001d048444 (encoding a protein related to sulfur starvation) and Zm00001d042228 (encoding a protein involved in the maintenance of undifferentiated cells), were only expressed in susceptible RILs. In addition, infected kernels of the resistant RILs were clearly different from those of the susceptible RILs in terms of transcript levels of genes involved in hormone biosynthesis and signaling pathways (Figure 2). In general, genes involved in strigolactone, brassinosteroid, and ethylene signaling were upregulated in the resistant RILs compared with the susceptible ones, while genes involved in the phytosulfokine, jasmonic acid, and salicylic acid signaling pathways were downregulated. Genes involved in auxin biosynthesis were downregulated, and those involved in gibberellin deactivation and auxin signaling repression and deactivation were upregulated in the resistant RILs. Alongside these patterns of gene expression, the resistant RILs showed changes in the transcript levels of genes implicated in the cell cycle, RNA metabolic processes, proteasome-ubiquitination system, post-translational modification of proteins, respiration, and primary and secondary metabolism. Some proteins encoded by these genes were strongly connected. Out of the proteins encoded by the 364 DEGs, 55 were connected with 77 edges (Figure 3). The interconnected proteins included those involved in breakdown of storage proteins, protein transport, proteasomal degradation of damaged or unneeded proteins, L-asparagine synthesis, cell division and differentiation, and RNA metabolism.

**FIGURE 4:**
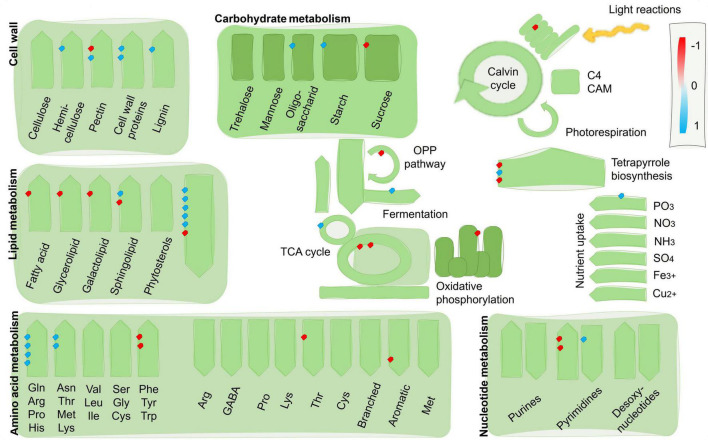
Distribution of DEGs between resistant and susceptible inbred bulks visualized with MapMan. Genes up and down-expressed in resistant vs. susceptible inbred bulks are in blue and red, respectively.

**FIGURE 5:**
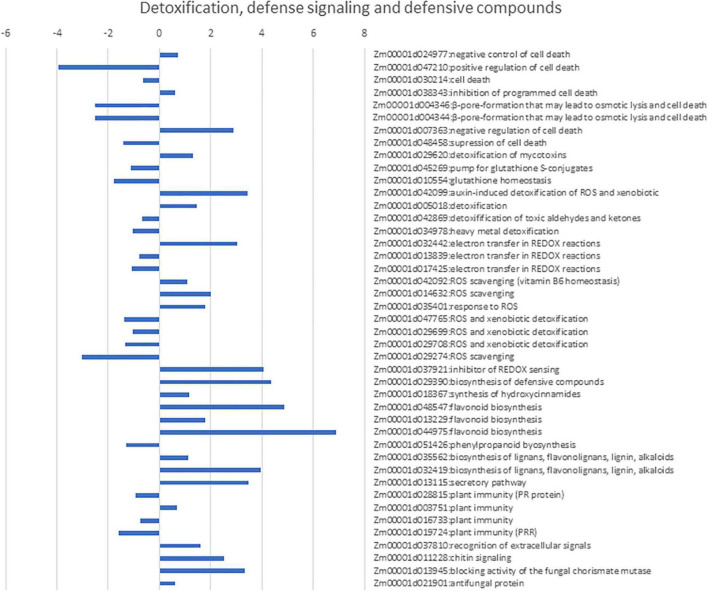
Expression Log_2_ fold change between inbred bulks resistant and susceptible to *Fusarium* ear rot and kernel contamination with fumonisins for differentially expressed genes (DEGs) related to detoxification, defense signaling, and biosynthesis of defensive compounds.

**FIGURE 6:**
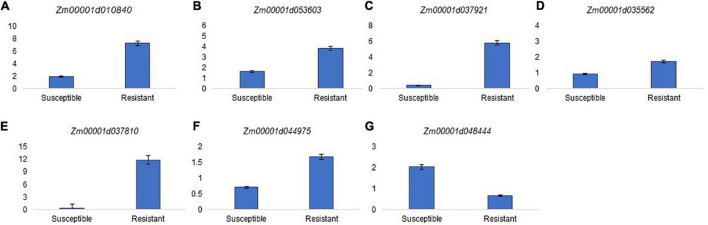
qRT-PCR expression levels of susceptible and resistant inbred bulks for seven genes **(A–G)**. Each bar represents the normalized (referred to *gap* gene expression) expression level of the gene. Student’s *t*-tests were performed for comparing inbred bulk expressions, and differences between resistant and susceptible inbred bulks were significant at *p* < 0.05 for the seven genes.

Compared with the susceptible RILs, the resistant RILs showed downregulation of genes involved in cell proliferation and upregulation of genes involved in cell extension and differentiation, especially those related to cell wall biosynthesis and modification (Figure 4). The resistant RILs also showed upregulation of genes involved in amino acid biosynthesis, lipid catabolism, and respiratory electron transport, and downregulation of genes related to sink metabolism and glyoxylate and the tricarboxylic acid (TCA) cycle. Specifically, the resistant lines showed upregulation of genes implicated in fungal defense signaling and biosynthesis of secondary metabolites likely involved in defense but not genes involved in programmed cell death (Figure 5). The qRT-PCR results confirmed the RNA-seq results in that the transcripts of genes Zm00001d010840, Zm00001d053603, Zm00001d035562, Zm00001d037810, Zm00001d037921, and Zm00001d044975 were significantly more abundant in the resistant bulks than in the susceptible ones; and vice versa for Zm00001d048444 (Figure 6).

### Differential Allelic Fixation

One or more SNPs were detected between the resistant and susceptible bulks in 37 DEGs across chromosomes 1, 3, 5, 6, 8, and 9; INDELs with presence/absence in resistant/susceptible bulks or vice versa were detected in 112 DEGs located across all the chromosomes (Supplementary Data Sheet 3). We detected SNPs and INDEL polymorphisms in 3, 4, 13, and 4 DEGs located in the confidence intervals of QTL for FER and/or fumonisin content at bins 4.07–4.1, 6–6.01, 6.04–6.05, and 8.05–8.08, respectively (Table 2).

**TABLE 2:** Candidate genes (more probable in bold) for QTL for fumonisin content and/or FER referred to the reference genome B73 RefGen_v4.

Gene	Chromosome	Position (bp)	*p*-value^1^	Log2FC^2^	Probable function
Zm00001d052080	4	178,783,005	0.03088099	−1.03	ROS scavenging
**Zm00001d053603**	**4**	**236,105,402**	**5.33E−05**	**1.02**	**Lipid transport**
Zm00001d053897	4	243,335,691	0.00261024	1.78	Ubiquitination-poteasesystem
**Zm00001d035562**	**6**	**33,367,726**	**3.28E−06**	**1.14**	**Secondary metabolism**
Zm00001d035621	6	37,468,979	0.00964222	3.63	Protein catabolism
Zm00001d035879	6	58,287,125	0.00445694	1.80	Electron transfer in respiratory metabolism
Zm00001d035960	6	62,647,186	0.0008752	0.78	Kernel development
Zm00001d037448	6	125,639,761	0.0116348	0.92	Ribosomal biogenesis
Zm00001d037480	6	126,663,869	0.01831076	0.87	Photoassimilate partitioning in sink tissue
Zm00001d037562	6	129,543,822	0.03088099	1.15	Negative regulation of cell proliferation
Zm00001d037722	6	135,231,059	0.02229742	2.64	Kernel morphogenesis
Zm00001d037724	6	135,240,118	0.04526894	**−**0.73	Degradation of gibberellins
Zm00001d037766	6	136,762,146	0.00690881	0.88	Positive regulator of auxin transport
**Zm00001d037810**	**6**	**138,532,222**	**2.29E−11**	**1.59**	**Recognition of extracellular signals**
Zm00001d037832	6	139,093,585	0.03641712	0.58	Transcription initiation
Zm00001d037890	6	141,204,831	0.00018441	4.27	Cell wall modification
Zm00001d037902	6	141,549,583	0.00043734	**−**0.95	Ubiquitination-poteasesystem
**Zm00001d037921**	**6**	**142,003,418**	**2.09E−14**	**4.05**	**REDOX sensing**
Zm00001d038039	6	145,912,636	3.25E−05	1.45	Vacuolar degradation of membrane proteins
Zm00001d038453	6	157,518,291	1.58E−05	0.70	Beta-alanine biosynthesis
**Zm00001d010840**	**8**	**129,739,531**	**1.35E−06**	**1.55**	**Lipid catabolism**
Zm00001d011228	8	143,868,837	0.04118359	2.53	Chitin signaling
Zm00001d011244	8	144,396,134	0.00114198	3.69	Strigolactone signaling
Zm00001d012353	8	172,918,437	4.28E−05	0.82	Ribosomal biogenesis

*^1^False discovery rate (FDR)-adjusted p-value of the expression difference between resistant and susceptible inbred bulks to kernel infection by F. verticillioides and fumonisin contamination.*

*^2^Log_2_ of the expression fold change (resistant inbred bulk/susceptible inbred bulk).*

## Discussion

### Genomics of *Fusarium* Ear Rot and Kernel Contamination With Fumonisins

This mapping population showed genetic variability for FER and fumonisin content in the kernels, with moderate heritability values for both traits. The heritability estimates for FER were in the range of values recently estimated for other bi-parental mapping populations, while those for fumonisin content were higher (Morales et al., 2019). A wide genetic variability for FER and fumonisin content was expected in this mapping population, because it was derived from the cross between two inbreds with contrasting values for FER and fumonisin content, with A637 being more resistant to FER and having lower fumonisin content than EP42 (Santiago et al., 2013).

Consistent with previous studies, the phenotypic and genotypic correlation coefficients between FER and fumonisin content were moderate to high. This suggested that, if selecting by phenotype, FER would be preferable to fumonisin content as the selection trait, because the former is less laborious and expensive to evaluate (Bolduan et al., 2009; Löffler et al., 2010, 2011; Samayoa et al., 2019). In addition, neither significant phenotypic nor genotypic correlations were found between FER and days to silking, indicating that phenological differences among the inbreds would not affect differences in FER among the RILs. In contrast, fumonisin accumulation may be affected by kernel humidity or other environmental conditions, as a previous study has shown that fumonisin contamination risk began earlier in plants sown later (Cao et al., 2013). Nevertheless, as expected based on the results of previous studies, the different performance of the RILs across environments greatly contributed to phenotypic differences among the RILs for FER, providing further evidence that molecular markers would be more useful than phenotypic values in selection programs (Santiago et al., 2020).

Most of the genetic variations in FER and fumonisin content could not be explained by the detected QTLs. No single QTL explained more than 10% of the phenotypic variability for FER or fumonisin content. Transgressive segregation was common, because almost half of the individuals in the mapping population had FER values lower than that of the resistant parent, A637. Transgressive segregation could result from the complementarity of favorable alleles from different parents but may also be indicative of additive × additive interaction effects. According to these two non-exclusive hypotheses, previous studies have shown that favorable alleles for FER do not always come from the resistant parent, and that there are QTLs with significant epistatic effects for reduced fumonisin levels (Robertson-Hoyt et al., 2006; Butron et al., 2015; Maschietto et al., 2017; Morales et al., 2019).

Overall, the results indicate that resistance to FER and reduced fumonisin accumulation are highly polygenic traits, and that the genetic variation in FER and fumonisin accumulation in the inbred lines is due to many QTLs with additive and additive × additive minor effects (Santiago et al., 2020). Therefore, the value of selection by QTL-linked markers will be limited because most genetic variability is not captured by QTL models because of the highly polygenic nature of the two traits (resistance to FER and reduced fumonisin contamination). However, selection gains would occur if genomic selection for the polygenic background for the target trait is performed alongside introgression of resistance alleles. In this context, Guo et al. (2020) showed that the prediction accuracy of genomic selection for FER was improved using trait-related markers. In this study, although the proportion of phenotypic variability in FER explained by each QTL was low, the QTLs were stable across environments and were moderately reliable, because two of them occurred in more than half of the CV runs. Therefore, molecular markers flanking QTLs for FER on chromosomes 4 and 8 could be included as fixed effects in models to predict genotypic values for genomic selection, although previously, QTL mapping using higher marker density would be implemented to narrow down the confidence intervals of the QTLs. In addition, the genomic regions detected in this study as relevant for resistance to FER and/or to contamination with fumonisins (at bins 4.07–4.1, 6.00–6.01, 6.04–6.05, and 8.05–8.08) have been previously highlighted in QTL mapping studies using bi-parental populations (Robertson-Hoyt et al., 2006; Maschietto et al., 2017; Morales et al., 2019; Wu et al., 2020) and in genome-wide association studies (Chen et al., 2016; Coan et al., 2018; Butron et al., 2019; Samayoa et al., 2019; Guo et al., 2020; Wu et al., 2020). Moreover, a QTL for FER found at bin 4.08 in a previous study was confirmed using near-isogenic lines (NILs) (Wu et al., 2020). It has been suggested that the region at 4.07–4.08 is a genomic hotspot for maize resistance to multiple pathogens and to mycotoxin accumulation, because QTLs related to eight different diseases and a validated QTL for aflatoxin content co-localize in that region (Wisser et al., 2006; Mideros et al., 2014). Therefore, this region deserves special attention, because it may contain genes related to resistance to multiple diseases across diverse genetic backgrounds. At bin 8.07, Samayoa et al. (2019) found an SNP significantly associated with fumonisin accumulation and proposed Zm00001d012327 (putative *ubiquitin-like-specific protease 2B* gene) as the candidate gene.

### Pathways Involved in *Fusarium* Infection

Quantitative trait loci studies not only identify markers for use in marker-assisted selection programs but also represent an intermediate step in locating genes involved in the inheritance of a trait. Because it is very difficult to find genes involved in resistance to FER and/or to fumonisin accumulation *via* QTL mapping, other approaches could be implemented to find genes related to these traits. Previous studies have detected DEGs between two unrelated inbred lines with contrasting resistance to FER in control plants and those inoculated with *F. verticillioides*. DEGs have been proposed as candidate genes for resistance; but differences in genetic backgrounds of inbred lines may have biased candidate gene identification in those studies (Lanubile et al., 2010, 2012a,b, 2014, 2017; Yuan et al., 2012, 2013; Campos-Bermudez et al., 2013; Yao et al., 2020). Our approach overcomes this problem, because we performed BSR, which combines BSA and RNA-seq. This minimizes biases due to background differences as long as each bulk contains individuals that are identical in one or more genomic regions implicated in a particular trait but arbitrary in all unlinked regions (Michelmore et al., 1991). Although BSA is more often used to map genes involved in monogenic inherited traits, it can be also a useful tool for mapping genes involved in quantitative traits such as resistance to FER and kernel fumonisin contamination (Liu et al., 2012; Du et al., 2017; Hou et al., 2018; Hao et al., 2019; Qin et al., 2020).

We detected 364 DEGs in immature kernels (25 days after pollination) between two groups of recombinant RILs with contrasting values for FER and fumonisin content after inoculation with *F. verticillioides*. However, no GO categories were significantly enriched among the DEGs. Similarly, Lanubile et al. (2014) found that DEGs between resistant and susceptible inbreds to FER were distributed among all functional classes.

However, DEGs in some specific pathways were, generally, upregulated or downregulated in resistant RILs compared with susceptible ones. Several genes involved in hormone biosynthesis, signaling, and homeostasis were differentially expressed between resistant and susceptible RILs, in accordance with the finding of Zhou et al. (2020) that genes involved in hormone signaling were among the target genes of *F. verticillioides*-responsive miRNAs. The plant hormones jasmonic acid, salicylic acid, and ethylene are directly involved in plant defense and regulate plant defense responses by cross-talk with other hormones such as abscisic acid, auxins, cytokinins, gibberellins, and strigolactones (Kazan and Lyons, 2014). In this study, ethylene signaling was upregulated in resistant vs. susceptible RILs; meanwhile salicylic and jasmonic acid signaling were slightly downregulated.

Upregulation of ethylene signaling was accompanied by increased transcript levels of genes involved in strigolactone and brassinosteroid signaling. Kapulnik et al. (2011) showed that strigolactones and ethylene affect some developmental processes through a common regulatory pathway in which ethylene is epistatic to strigolactones. The coordination of the ethylene-brassinosteroid pathway could be responsible for balancing resistance and growth (Liu et al., 2017). Therefore, ethylene signaling may regulate the functions of strigolactones and brassinosteroids in resistance to FER and fumonisin accumulation in kernels. All the three hormones can negatively affect the growth of *Fusarium* species: strigolactones were shown to strongly inhibit the growth of *Fusarium oxysporum* and *Fusarium solani*; brassinosteroid was shown to enhance the resistance of barley to *Fusarium* diseases; and ethylene signaling was shown to affect the relative abundance of metabolites contributing to resistance to *Fusarium graminearum* in maize (Dor et al., 2011; Ali et al., 2013; Zhou et al., 2019).

The downregulation of genes involved in jasmonic acid signaling in the resistant RILs was not unexpected. Christensen et al. (2014) provided genetic evidence for the involvement of jasmonic acid in maize defense against *F. verticillioides*, while Lanubile et al. (2015) showed that maize resistance depends on early activation of LOX genes, even though the activation of LOX genes was stronger in susceptible inbred lines than in resistant ones 14 dpi.

Auxin biosynthesis and signaling were downregulated in the resistant RILs compared with the susceptible ones. Again, this was not surprising, because pathogens have developed strategies *via* their effector repertoire to either interfere, or hijack phytohormone pathways to induce host susceptibility. Pathogen-induced auxin biosynthesis or modulated auxin signaling has been associated with increased host susceptibility. In this context, Brauer et al. (2019) found that the auxin pathway was one of the few susceptibility-associated pathways that functioned during wheat kernel infection by *F. graminearum*.

Among DEGs between resistant and susceptible RILs, there were also genes involved in cell cycle, respiration, and primary and secondary metabolism, suggesting that resistant RILs make a growth–defense trade-off to optimize their fitness during *F. verticillioides* infection (Yao et al., 2020). According to a possible growth–defense trade-off, sink metabolic processes such as fatty acid and starch biosynthesis were downregulated as well as phytosulfokine signaling, which regulates cell proliferation and growth, and many other genes involved in cell division. However, genes related to cell expansion and differentiation were upregulated, especially those involved in cell wall biosynthesis and modification that could be controlled and organized, in part, by the cytoskeleton and mediated by auxin transport (Perrot-Rechenmann, 2010; Bashline et al., 2014). Lanubile et al. (2014) also reported that the response of one resistant inbred line to *F. verticillioides* infection included activation of genes related to lignin, a structural component of the plant cell wall. Induced systemic resistance (ISR) in maize is activated by the beneficial fungus *Trichoderma atroviride*, and this process involves reinforcement of plant cell walls, which reduces the development of disease after inoculation with *F. verticillioides* (Agostini et al., 2019).

In this study, glycolysis, tricarboxylic acid, and glyoxylate cycles were downregulated concomitantly with reduction in sink processes such as starch and fatty acid biosynthesis in the resistant RILs. However, genes involved in electron transfer during respiratory metabolism were upregulated, as were those involved in amino acid biosynthesis. This suggested that the electron transport chain could be activated toward amino acid synthesis under conditions of high TCA cycle flux (Fernie et al., 2004; Li et al., 2020). Ciasca et al. (2020) recently showed that levels of the amino acid tyrosine were significantly higher in an inbred line resistant to *F. verticillioides* infection than in its susceptible counterpart, and that the opposite was true for L-tryptophan.

In terms of plant defense pathways, genes directly involved in defense mechanisms were activated in the resistant RILs, while genes involved in cell death were repressed. In previous transcriptomic studies, activation of genes involved in secondary metabolism, such as those in shikimate, lignin, flavonoid, and terpenoid pathways, has been observed in resistant vs. susceptible genotypes, or in plants colonized by beneficial fungi (Lanubile et al., 2014; Agostini et al., 2019). In this study, one of the most strongly activated secondary pathways was the flavonoid branch of the phenylpropanoid pathway. This is consistent with reports that flavonoid compounds such as flavones, flavonols, and anthocyanins rather than hydroxycinnamic acids contribute to differences in FER resistance and fumonisin accumulation among maize cultivars (Venturini et al., 2016; Bernardi et al., 2018). The antioxidant properties of flavonoids could also explain why genes involved in reactive oxygen species scavenging and detoxification were, in general, downregulated in resistant RILs, although another study detected them among upregulated genes in resistant vs. susceptible inbred lines (Maschietto et al., 2016).

Overall, the increased secondary metabolism in resistant RILs could be supported by decreased sink metabolism and increased catabolic processes to produce substrates for secondary metabolism. However, some authors have highlighted more direct roles of plant protein and pectin degradation, for example, plant proteolytic systems are crucial for defense against pathogens, and oligogalacturonides are elicitors of plant defense responses (Baek and Choi, 2008; Lionetti, 2015). In addition, some DEGs encode proteins that may be involved in degradation of fungal defenses, for example, secretory lipases that degrade the fungal arsenal of lipids and specific glucanases that target the fungal cell wall (Lozovaya et al., 1998; Debona et al., 2017; Lee and Park, 2019).

### Candidate Genes

Besides giving an overview on differences in gene expression between resistant and susceptible inbred lines, RNA-seq analyses can help to uncover genomic regions involved in resistance. To decrease the probability of detecting false positive associations, only polymorphisms in DEGs resulting from fixation of one allele in resistant inbreds and fixation of the alternative allele in susceptible inbreds, along with INDEL polymorphisms (presence/absence in resistant/susceptible inbreds or vice versa), were used to map genomic regions likely associated with resistance. Resistant RILs presented resistant alleles for at least one marker located in the confidence interval of each QTL, while susceptible RIL lines carried susceptible alleles for at least one marker contained in the confidence interval of each QTL. DEGs with polymorphisms between resistant and susceptible inbreds were scattered across 43 bins of the 10 chromosomes, consistent with the highly polygenic nature of the studied traits (Zila et al., 2014; Samayoa et al., 2019).

The DEGs located within confidence intervals of detected QTLs and showing polymorphisms between resistant and susceptible RILs were identified as valuable candidate genes for those QTLs and deserve special attention. Differences in expression were especially noticeable for Zm00001d053603, Zm00001d035562, Zm00001d037810, Zm00001d037921, and Zm00001d010840 and were confirmed by qRT-PCR. Zm00001d053603 encodes an ABC transporter A family member involved in lipid transport, and Zm00001d010840 encodes triacylglycerol lipase-like 1, which is involved in degradation of oil bodies in seeds. The upregulation of these genes could be related to increased resistance to FER, because they would participate in mobilization of lipids from oil bodies to phytoalexin synthesis (Shimada et al., 2014). Similarly, Zm00001d035562, encoding a dirigent protein, 100502041, that yields optically active lignans from two molecules of coniferyl alcohol, could be implicated in resistance to FER through its involvement in lignan biosynthesis and/or other aspects of secondary cell wall biosynthesis (Corbin et al., 2018). Zm00001d037810 encodes a G-type lectin S-receptor-like serine/threonine-protein kinase, At2g19130. Lectin receptor-like kinases are cell surface receptors that play important roles in perceiving and processing signals that arrive in cells, such as fungi, because their extracellular domains can bind to fungal cell wall components (Vaid et al., 2013; Liu et al., 2018). Finally, Zm00001d037921 is orthologous to the *MWD9.13* gene in *Arabidopsis* and encodes an NF-κB inhibitor-like protein. In humans, the NF-κB family of transcription factors regulates the expression of hundreds of genes associated with cell proliferation, differentiation, and death, and immune responses (Golan-Goldhirsh and Gopas, 2014). In *Arabidopsis*, homologs of NF-κB transcription factors have been proposed to participate in redox sensing (Li et al., 2006). Therefore, polymorphisms in the gene encoding *NF-κB inhibitor-like protein* may interfere in oxidative response to *F. verticillioides* and contribute to resistance to FER and fumonisin accumulation, since increased oxidative stress favors mycotoxin biosynthesis (Picot et al., 2010). According to the hypothesis of reduced oxidative stress in resistant kernels, genes related to cell death were downregulated in the resistant compared with the susceptible RILs. These candidate genes should be validated in future studies.

## Conclusion

Functions of the DEGs between the resistant and susceptible inbred bulks suggest a growth–defense trade-off, because sink metabolic processes such as fatty acid and starch biosynthesis were downregulated, as well as phytosulfokine signaling and many other genes involved in cell division, while genes related to substances and processes of the secondary metabolism associated to resistance, such as those involved in cell wall biosynthesis and its adjustment, and flavonoid biosynthesis, were upregulated. Among the DEGs, five (Zm00001d053603, Zm00001d035562, Zm00001d037810, Zm00001d037921, and Zm00001d010840) were polymorphic between the resistant and susceptible bulks, were located within the confidence intervals of detected QTLs and showed large differences in transcript abundance between the resistant and susceptible bulks. Thus, they were identified as candidate genes for the detected QTLs.

## Data Availability Statement

The datasets presented in this study can be found in online repositories. The names of the repository/repositories and accession number(s) can be found below: http://hdl.handle.net/10261/258356, https://doi.org/10.20350/digitalCSIC/14486.

## Author Contributions

AB conceived the study. AC took care of the field and greenhouse experiments, data recording, and sample collection with the assistance of AB, RS, and RM. MF led all the molecular biology analyses. AB and MF performed the statistical analyses of data. AB and NG drafted the initial manuscript. NG edited the manuscript. All authors read and approved the final version of the manuscript.

## Conflict of Interest

The authors declare that the research was conducted in the absence of any commercial or financial relationships that could be construed as a potential conflict of interest.

## Publisher’s Note

All claims expressed in this article are solely those of the authors and do not necessarily represent those of their affiliated organizations, or those of the publisher, the editors and the reviewers. Any product that may be evaluated in this article, or claim that may be made by its manufacturer, is not guaranteed or endorsed by the publisher.

## Footnotes

1
http://bioinformatics.sdstate.edu/go/

2
https://string-db.org/
