# Supplementary figures and images for: Genomics and Pathways Involved in Maize Resistance to Fusarium Ear Rot and Kernel Contamination With Fumonisins
Source: Front Plant Sci. 2022 May 2;13:866478. doi: 10.3389/fpls.2022.866478 (PMC9108495; doi:10.3389/fpls.2022.866478)

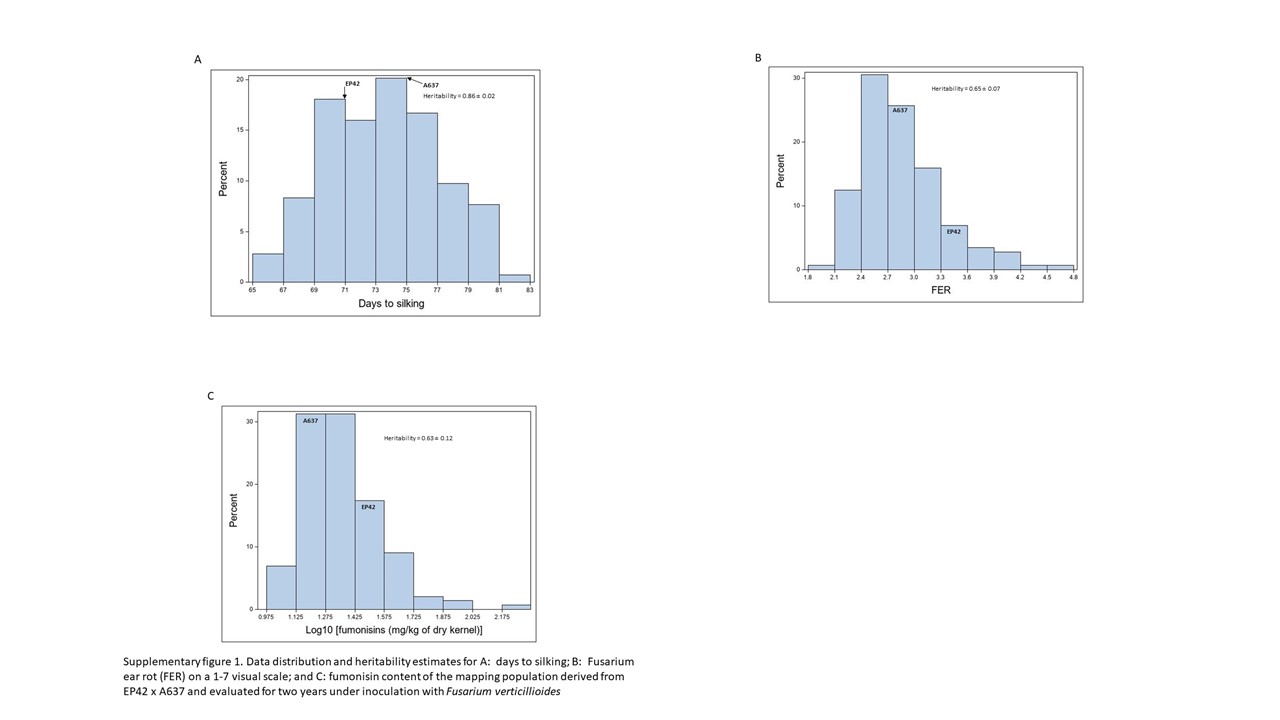

Supplement: Supplementary file 4 [file Image_1.JPEG]
