# Supplementary material for: Genomics and Pathways Involved in Maize Resistance to Fusarium Ear Rot and Kernel Contamination With Fumonisins
Source: Front Plant Sci. 2022 May 2;13:866478. doi: 10.3389/fpls.2022.866478 (PMC9108495; doi:10.3389/fpls.2022.866478)
Supplement: Supplementary file 5 [file Table_1.DOCX]

**Supplementary table 1. RNA-seq data quality summary**

| **Sample^1^** | **Raw reads^2^** | **Raw data^3^** | **Effective^4^** | **Error^5^** | **Q20(%)^6^** | **Q30(%)** | **GC(%)^7^** |
| --- | --- | --- | --- | --- | --- | --- | --- |
| A1 | 63684194 | 9.6 | 98.34 | 0.02 | 97.90 | 94.33 | 53.88 |
| A4 | 72810934 | 10.9 | 97.83 | 0.03 | 97.57 | 93.99 | 54.55 |
| A5 | 66817782 | 10.0 | 98.42 | 0.02 | 97.94 | 94.41 | 53.73 |
| A3 | 48215378 | 7.2 | 98.65 | 0.03 | 97.87 | 94.31 | 54.56 |
| A2 | 59626720 | 8.9 | 98.83 | 0.02 | 97.94 | 94.32 | 54.12 |
| A6 | 59526612 | 8.9 | 98.34 | 0.02 | 97.98 | 94.48 | 54.21 |

^1^Sample: sample name (A1-A3 for RIL resistant bulks and A4-A6 for susceptible RIL bulks).
^2^Raw reads: total amount of reads of raw data.

^3^Raw data: (raw reads) * (sequence length =150 bp), calculated in Gbp.

^4^Percentage of effective reads: (clean reads/raw reads)*100
^5^Error: base error rate in percentage
^6^Q20, Q30: base count of Phred value > 20 or 30*100 / Total base count
^7^GC: G & C base count *100/ total base count
