# Supplementary material for: Genomics and Pathways Involved in Maize Resistance to Fusarium Ear Rot and Kernel Contamination With Fumonisins
Source: Front Plant Sci. 2022 May 2;13:866478. doi: 10.3389/fpls.2022.866478 (PMC9108495; doi:10.3389/fpls.2022.866478)
Supplement: Supplementary file 7 [file Table_3.DOCX]

Supplementary table 3. Analysis of variance of the maize recombinant inbred lines (RIL) population derived from EP42 x A637 evaluated for days to silking, Fusarium ear rot (FER) and kernel fumonisin content under inoculation with Fusarium verticillioides in two years.

|  |  | Days to silking | |  | FER | |  | Fumonisin content | |
| --- | --- | --- | --- | --- | --- | --- | --- | --- | --- |
| Source of variation |  | Z-value | *p-*value |  | Z-value | *p-*value |  | Z-value | *p-*value |
| Year |  | 0.70 | 0.2421 |  | 0.68 | 0.2483 |  | 0.54 | 0.2948 |
| Replication (Year) |  | - | - |  | - | - |  | 0.78 | 0.2165 |
| Block (Year*Replication) |  | 3.85 | <0.0001 |  | 1.48 | 0.0692 |  | 1.33 | 0.0914 |
| RIL |  | 7.07 | <0.0001 |  | 4.87 | <0.0001 |  | 2.92 | 0.0018 |
| Year*RIL |  | 3.49 | 0.0002 |  | 2.36 | 0.0091 |  | 1.28 | 0.1005 |
| Error |  | 10.51 | <0.0001 |  | 9.15 | <0.0001 |  | 9.39 | <0.0001 |
